# “Serial ferritin titer” monitoring in COVID-19 pneumonia: valuable inflammatory marker in assessment of severity and predicting early lung fibrosis — prospective, multicentric, observational, and interventional study in tertiary care setting in India

**DOI:** 10.1186/s43162-022-00163-3

**Published:** 2022-10-12

**Authors:** Shital Patil, Gajanan Gondhali, Abhijit Acharya

**Affiliations:** 1 Pulmonary Medicine, MIMSR Medical College, Latur, India; 2 Internal Medicine, MIMSR Medical College, Latur, India; 3 Department of Pathology, MIMSR Medical College, Latur, India

**Keywords:** COVID-19 pneumonia, Ferritin, Oxygen saturation, Inflammatory marker

## Abstract

**Introduction:**

The COVID-19 pneumonia is a heterogeneous disease with variable effect on lung parenchyma, airways, and vasculature leading to long-term effects on lung functions.

**Materials and methods:**

Multicentric, prospective, observational, and interventional study conducted during July 2020 to May 2021, in the MIMSR Medical College and Venkatesh Hospital Latur India, included 1000 COVID-19 cases confirmed with RT-PCR. All cases were assessed with lung involvement documented and categorized on HRCT thorax, oxygen saturation, inflammatory marker, ferritin at entry point, and follow-up during hospitalization. Age, gender, comorbidity, and use of BIPAP/NIV and outcome as with or without lung fibrosis as per CT severity were key observations. CT severity scoring is done as per universally accepted standard scoring tool as score < 7 as mild, 7–14 as moderate, and score > 15 as severe affection of the lung. Statistical analysis is done by using chi-square test.

**Observations and analysis:**

In study of 1000 COVID-19 pneumonia cases, age (< 50 and > 50 years) and gender (male versus female) have significant association with ferritin in predicting severity of COVID-19 pneumonia (*p* < 0.00001) and (*p* < 0.010), respectively. CT severity score at entry point with ferritin level has significant correlation in severity scores < 8, 8–15, and > 15 documented in normal and abnormal ferritin level as in 190/110, 90/210, and 40/360, respectively (*p* < 0.00001). Ferritin level has significant association with duration of illness, i.e., *DOI* < 7 days, 8–15 days, and > 15 days of onset of symptoms documented normal and abnormal ferritin levels in 30/310, 160/300, and 130/70 cases, respectively (*p* < 0.00001). Comorbidity as diabetes mellitus, hypertension, COPD, IHD, and obesity has significant association in COVID-19 cases with normal and abnormal ferritin level respectively (*p* < 0.00001). Ferritin level has significant association with oxygen saturation in COVID-19 pneumonia cases; cases with oxygen saturation > 90%, 75–90%, and < 75% are observed as normal and abnormal ferritin level in 110/100, 150/340, and 60/240 cases, respectively (*p* < 0.00001). BIPAP/NIV requirement during the course of COVID-19 pneumonia in critical care setting has significant association with ferritin level; cases received BIPAP/NIV during hospitalization were documented normal and abnormal ferritin level in 155/445 and 165/235 cases, respectively (*p* < 0.00001). Timing of BIPAP/NIV requirement during course of COVID-19 pneumonia in critical care setting has significant association with ferritin level; cases received BIPAP/NIV at entry point < 1 day, 3–7 days, and after 7 days of hospitalization were documented significance in fourfold raised ferritin level in 110/70, 150/160, and 30/80 cases, respectively (*p* < 0.00001). Follow-up of ferritin titer during hospitalization as compared to entry point abnormal ferritin has significant association in post-COVID lung fibrosis (*p* < 0.00001). Follow-up of ferritin titer during hospitalization as compared to entry point normal ferritin has significant association in post-COVID lung fibrosis (*p* < 0.00001).

**Conclusion:**

Ferritin is easily available, sensitive and reliable, cost-effective, and universally acceptable inflammatory marker in COVID-19 pandemic. Ferritin has very crucial role in COVID-19 pneumonia in predicting severity of illness and assessing response to treatment during hospitalization. Follow-up of ferritin titer during hospitalization and at discharge can be used as early predictor of post-COVID lung fibrosis.

## Introduction

The current pandemic of coronavirus disease-2019 (COVID-19) caused by SARS-CoV-2 originally emerged from China but has since then confirmed cases of 274,628,461 and deaths 5,358,978 worldwide and 34,752,164 confirmed cases and 478,007 total deaths in India alone [1]. The International Federation of Clinical Chemistry and Laboratory Medicine (IFCC) task force on COVID-19 has been established to synthesize up-to-date information on the epidemiology, pathogenesis, and laboratory diagnosis and monitoring of COVID-19, as well as to develop practical recommendations on the use of molecular, serological, and biochemical tests in disease diagnosis and management [2, 3].

COVID-19 pneumonia is a heterogeneous disease with variable effect on lung parenchyma, airways, and vasculature leading to long-term effects on lung functions. Although the lung is the primary target organ involvement in coronavirus disease-19 (COVID-19), many patients were shown pulmonary and extrapulmonary manifestations of diseases variably during the first and second wave, which occurred as resultant pathophysiological effects of immune activation pathway and direct virus-induced lung damage. In COVID-19 pneumonia, pathophysiology constitutes different pathways like immune activation, inflammatory, thrombogenic, and direct viral affection to lungs and extrapulmonary tissues [4, 5].

Ferritin is a highly ubiquitous iron-binding protein first isolated in 1937 from horse spleen; since then, its isolation methodology and role as acute phase reactant and role as marker of inflammation have been evolved over decades. Various inflammatory markers like ferritin, LDH, CRP, IL-6, and D-dimer have been evaluated in this pandemic, and now robust data is available regarding its usefulness in analyzing severity, decision-making in critical cases, assessing response to interventions, and predicting outcome. Cytokine syndrome is defined as “A group of conditions sharing same pathological mechanisms with different etiologies, causing massive release of pro-inflammatory cytokines resulting into aberrant activation of immune and coagulation systems” [6]. Cytokine storms have direct association with raised ferritin level, and indirectly, it will help in predicting ongoing inflammatory surge resulting in cytokine storm. Cytokine storm is the most dreadful event in pathophysiology of COVID-19 pneumonia, and ultimately, it will lead to either direct cytokine-induced lung injury manifesting as ALI/ARDS or extrapulmonary systemic secondary hemophagocytic lymphohistiocytosis [7]. Studies have documented significantly raised ferritin with other inflammatory markers in COVID-19 pneumonia [8], and now, COVID-19 has been included in conditions causing hyperferritinemia [7].

Ferritin analysis is found to be very crucial in this COVID-19 pneumonia, apart from routine inflammatory marker and its usefulness as a marker of underlying immunosuppression [9]. Additionally, it is useful in predicting severity of illness in patients suffering with comorbidity as diabetes mellitus and in geriatric cases and marker of increased morbidity in these cases [10–12].

In present study, we have utilized ferritin as basic marker in laboratory panel workup in all COVID patients and analyzed as core marker during follow-up in all admitted patients to assess response to therapy and predictor of post-COVID fibrosis as dismal outcome of this pandemic of pneumonia in tertiary care setting.

## Materials and methods

Multicentric, prospective, observational, and interventional study, conducted during July 2020 to May 2021, in MIMSR Medical College and Venkatesh Hospital, Latur, India, included 1000 COVID-19 cases confirmed with RT-PCR, to find out the role of ferritin in predicting severity of illness and assessing response to therapy and outcome as post-COVID fibrosis in diagnosed COVID-19 pneumonia cases admitted in critical care unit. Total 1000 cases were enrolled in study after IRB approval and written informed consent of patient.

### Inclusion criteria

1. All COVID-19 pneumonia cases above 18 years of age, admitted in indoor unit, have been enrolled in the study.
2. COVID-19 pneumonia irrespective of CT severity was enrolled in the study.
3. COVID-19 pneumonia irrespective oxygen saturation was enrolled in the study.
4. COVID-19 pneumonia with comorbidity like diabetes mellitus, IHD, CVD, CKD, and COPD was enrolled in the study.
5. COVID-19 pneumonia cases willing to undergo follow-up of ferritin analysis during hospitalization were enrolled in the study.

### Exclusion criteria

1. COVID-19 pneumonia cases not willing to participate in study
2. COVID-19 pneumonia cases not willing to undergo follow-up on ferritin analysis
3. COVID-19 pneumonia cases want to be discharge against medical advice before clinical recovery from hospital
4. COVID-19 pneumonia below 18 years of age

### All study cases undergo the following assessment before enrolling in the study:

1. COVID-19 RT-PCR test
2. COVID-19 rapid antigen tests if positive were included, and if antigen test is negative, then all tests are subjected to COVID-19 RT-PCR test.
3. HRCT thorax to assess severity of lung involvement and categorized as mild if score < 7, moderated if score 8–15, and severe if score > 15 or 15–25
4. Clinical assessment as vital parameters like heart rate, respiratory rate, blood pressure, and documentation of respiratory adventitious sounds
5. Laboratory parameters — hemoglobin, renal functions, blood sugar level, kidney functions, and ECG
6. Hematological parameters like total white blood cell counts, platelet count, and repeated whenever required if initial documentation is abnormal. Normal and abnormal parameter readings were considered as per pathological laboratory standard.
7. Viral inflammatory markers like CRP, ferritin, LDH, and IL-6 assessed at entry point and repeated whenever required during course of illness. Normal and abnormal parameter readings were considered as per pathological laboratory standard.
8. Entry point ferritin titer was utilized as assessment tool of severity of illness with clinical parameters.
9. If ferritin analysis was normal at entry point, then ferritin titer was repeated on the day of discharge from hospital or done during hospitalization if clinical course deteriorates.
10. If ferritin analysis was abnormal at entry point, we repeated on every 72 h as follow-up to assess severity, progression of illness, and also titer level utilized to assess response to medical treatment.

### Methodology of ferritin titer assessment

#### Principle: sandwich immunoluminometric assay

Use an anti-ferritin monoclonal antibody to label ABEI, and use another monoclonal antibody to label FITC. Sample calibrator or control is mixed thoroughly with FITC label and nano magnetic microbeads in a cuvette incubated at 37 °C, and then cycle was wash for 1 time. Then, add ABEI label and incubation and form a sandwich and then wash for the 2nd time. Subsequently, the starter reagents are added, and a flash chemiluminescent reaction is initiated. The light signal is measured by a photomultiplier as RLU within 3 s and is proportional to the concentration of ferritin present in samples.

#### Interpretation of results with reference values

1. Male: 14–250 ng/ml
2. Female: Age < 45 years old 6–160 ng/ml and age ≥ 45 years old 5–200 ng/ml
3. Results may differ between laboratories due to variations in population and test method. Each laboratory should establish its own reference range.

#### Interpretation of results

1. Negative: Values within normal limit as per gender
2. Positive: Value above reference range as per gender
3. Significant: Twofold raised value as per gender
4. Highly significant: Fourfold raised as per gender
5. Follow-up significance: Values raised or decreased in two- to fourfold change as per gender

The statistical analysis was done using chi-squared test. Significant values of *χ*^2^ were seen from probability table for different degrees of freedom required. *P*-value was considered significant if it was below 0.05 and highly significant in case if it was less than 0.001.

### Observations and analysis

In present study, 1000 COVID-19 pneumonia cases are confirmed by COVID-19 RT-PCR, males were 650/1000 and females were 350/1000, age > 50 were 600 cases, and age < 50 were 400 cases.

CT severity score at entry point with ferritin level has significant correlation in COVID-19 pneumonia cases; scores < 8, 8–15, and > 15 documented normal and abnormal ferritin level as in 190/110, 90/210, and 40/360, respectively, of total 1000 study cases (*p* < 0.00001) (Table 1).

**Table 1:** Correlation of CT severity (at entry point) and ferritin in covid-19 cases (*n* = 1000)

CT severity	Normal ferritin (*n* = 320)	Abnormal ferritin level (*n* = 680)	Analysis
< 8 score (*n* = 300)	190	110	Chi test value 224.87 *p* < 0.00001
9–15 (*n* = 300)	90	210	
> 15 (*n* = 400)	40	360	

Ferritin level has significant association with duration of illness in COVID-19 pneumonia cases; *DOI* < 7 days, 8–15 days, and > 15 days of onset of symptoms documented normal and abnormal ferritin levels in 30/310, 160/300, and 130/70 cases, respectively (*p* < 0.00001) (Table 2).

**Table 2:** Duration of illness (DOI) at entry point during hospitalization and ferritin level in COVID-19 pneumonia cases (*n* = 1000)

Duration of illness	Normal ferritin (*n* = 320)	Abnormal ferritin (*n* = 680)	Analysis
< 7 days (*n* = 340)	30	310	Chi test value 185.65 *p* < 0.00001
8–15 days (*n* = 460)	160	300	
> 15 days (*n* = 200)	130	70	

Significant association in ferritin and COVID-19 pneumonia has been documented with variables like age, gender, diabetes mellitus, IHD, hypertension, COPD, and obesity (*p* < 0.00001) (Table 3).

**Table 3:** Other variables and ferritin level in COVID-19 pneumonia cases (*n* = 1000)

COVID-19 RT-PCR positive (*n* = 1000)	Ferritin level normal (*n* = 320)	Ferritin level abnormal (*n* = 680)	Chi test value and *p*-value
Age > 50 years (*n* = 600)	140	460	*χ*^2^ = 51.77 *p* < 0.00001
Age < 50 years (*n* = 400)	180	220	
Male gender (*n* = 650)	190	460	*χ*^2^ = 6.5 *p* < 0.010
Female gender (*n* = 350)	130	220	
Diabetes mellitus (*n* = 600)	150	450	*χ*^2^ = 33.77 *p* < 0.00001
Without diabetes (*n* = 400)	170	230	
Hypertension (*n* = 210)	160	50	*χ*^2^ = 238.55 *p* < 0.00001
Without hypertension (*n* = 790)	160	630	
COPD (*n* = 150)	100	50	*χ*^2^ = 97.46 *p* < 0.00001
Without COPD (*n* = 850)	220	630	
IHD (*n* = 200)	110	90	*χ*^2^ = 60.77 *p* < 0.00001
Without IHD (*n* = 800)	210	590	
Obesity (*n* = 160)	20	140	*χ*^2^ = 33.28 *p* < 0.00001
Without obesity (*n* = 840)	300	540	

Ferritin level has significant association with oxygen saturation in COVID-19 pneumonia cases; cases with oxygen saturation > 90%, 75–90%, and < 75% are observed as normal and abnormal ferritin level in 110/100, 150/340, and 60/240 cases, respectively (*p* < 0.00001) (Table 4).

**Table 4:** Oxygen saturation at entry point and ferritin level in COVID-19 pneumonia cases (*n* = 1000)

Oxygen saturation	Normal ferritin level (*n* = 320)	Abnormal ferritin level (*n* = 680)	Analysis
> 90% (*n* = 210)	110	100	Chi test value 60.37 *p* < 0.00001
75–90% (*n* = 490)	150	340	
< 75% (*n* = 300)	60	240	

BIPAP/NIV requirement during course of COVID-19 pneumonia in critical care setting has significant association with ferritin level; cases received BIPAP/NIV during hospitalization were documented normal and abnormal ferritin level in 155/445 and 165/235 cases, respectively (*p* < 0.00001) (Table 5).

**Table 5:** Correlation of BIPAP use with ferritin level in COVID-19 pneumonia cases (*n* = 1000)

BIPAP/NIV	Normal ferritin (*n* = 320)	Abnormal ferritin level (*n* = 680)	Analysis
BIPAP/NIV required (*n* = 600)	155	445	Chi test value 26.21 *p* < 0.00001
BIPAP/NIV not required (*n* = 400)	165	235	

Timing of BIPAP/NIV requirement during course of COVID-19 pneumonia in critical care setting has significant association with ferritin level; cases received BIPAP/NIV at entry point < 1 day, 3–7 days, and after 7 days of hospitalization were documented significance in fourfold raised ferritin level in 110/70, 150/160, and 30/80 cases, respectively (*p* < 0.00001) (Table 6).

**Table 6:** BIPAP/NIV initiation time at entry point and ferritin level COVID-19 pneumonia cases (*n* = 600)

BIPAP used (*n* = 600) with duration of illness	Abnormal ferritin level (*n* = 290)	Fourfold raised ferritin level (*n* = 310)	Analysis
Entry point < 1 day (*n* = 180)	110	70	Chi test value 31.30 *p* < 0.00001
3–7 days (*n* = 310)	150	160	
After 7 days (*n* = 110)	30	80	

Follow-up of ferritin titer during hospitalization as compared to entry point abnormal ferritin has significant association in post-COVID lung fibrosis (*p* < 0.00001), i.e., ferritin at entry point to fourfold raised cases in the presence or absence of pulmonary fibrosis was 40/170 and 360/110 cases, respectively (Table 7).

**Table 7:** Abnormal ferritin level at entry point (*n* = 680) and follow-up and its correlation with post-COVID lung fibrosis

Post-COVID pneumonia fibrosis	Ferritin titer increased/abnormal at entry point (*n* = 400)	Ferritin titer fourfold increased during follow-up (*n* = 280)	Analysis
Pulmonary fibrosis present (*n* = 210)	40	170	Chi test value 198.45 *p* < 0.00001
Pulmonary fibrosis absent (*n* = 470)	360	110	

Follow-up ferritin titer during hospitalization as compared to entry point normal. Ferritin has significant association in post-COVID lung fibrosis (*p* < 0.00001), i.e., ferritin at entry point to fourfold raised cases in the presence or absence of pulmonary fibrosis was 5/35 and 115/165 cases, respectively (Table 8).

**Table 8:** Normal ferritin level (*n* = 320) at entry point and follow-up and its correlation with post-COVID lung fibrosis

Post-COVID pneumonia fibrosis	Ferritin normal at entry point and remained less than fourfold (*n* = 120)	Ferritin titer fourfold increased during follow-up (*n* = 200)	
Pulmonary fibrosis present (*n* = 40)	5	35	Chi test value 12.19 *p* < 0.00048
Pulmonary fibrosis absent (*n* = 280)	115	165	

## Discussion

### Correlation of CT severity (at entry point) and ferritin in COVID-19 cases

In present study, CT severity score at entry point with ferritin level has significant correlation in COVID-19 pneumonia cases; scores < 8, 8–5, and > 15 documented normal and abnormal ferritin level as in 190/110, 90/210, and 40/360, respectively, of total 1000 study cases (*p* < 0.00001).

We have documented that as CT severity increases, inflammatory marker ferritin also increases, and significant number of mild cases was also having abnormal ferritin level. Authors Zhou F. et al. [4] and Berna Kömürcüoğlu et al. [13] documented similar observation in their study and mentioned that radiological severity as per HRCT thorax has correlation with inflammatory markers like ferritin, CRP, D-dimer, and lymphopenia and observed their prognostic role in COVID-19 pneumonia. Author Dahan et al. [14] observed role of ferritin as “severity predictor” in their study with raised ferritin level in advanced CT severity cases. Authors Allen M. et al. [15], Zhou P. et al. [16], and Alroomi M. et al. [17] observed similar observation as positive association with CT severity and as CT severity increases which is the marker of more lung parenchymal inflammation and resulted in increased inflammatory burden documenting increased ferritin. Authors Del Valle et al. [18] and Huang S. et al. [19], Montecino-rodriguez E. et al. [20], Dhont S. et al. [21], and Wu C. et al. [22] observed that ferritin level is very well collaborated with CT severity and is highly significant in very severe group of COVID-19 pneumonia cases.

We have observed CT severity as the best visual marker of severity of COVID-19 pneumonia which can be correlated with inflammatory markers like ferritin, CRP, IL-6 LDH, D-dimer and lymphopenia, and lymphocyte platelet ratio, and it will help in triaging cases in casualty and help in targeting interventions in indoor units accordingly to have successful treatment outcome. Fox S. E. et al. [23] documented in autopsy series in New Orleans regarding high ferritin level in cases with advanced pneumonia showing necrosis and hyaline membrane formation on histopathology of lung specimens.

### Duration of illness (DOI) at entry point during hospitalization and ferritin level in COVID-19 pneumonia cases (*n* = 1000)

In present study, ferritin level has significant association with duration of illness in COVID-19 pneumonia cases; *DOI* < 7 days, 8–15 days, and > 15 days of onset of symptoms documented normal and abnormal ferritin levels in 30/310, 160/300, and 130/70 cases, respectively (*p* < 0.00001).

Although ferritin is raised in COVID-19 pneumonia, we have documented that proportionate number of cases with duration of illness < 1 week or 7 days and many cases with duration of illness > 2 weeks or 15 days were having normal ferritin level, while pneumonia cases between 7 and 14 days of illness were having abnormal or raised ferritin level. Rationale for observation is not known, may be inflammatory response pattern is different, and we have correlated ferritin pattern with other inflammatory markers like CRP, IL-6, and LDH and documented that these two markers raised parallel to ferritin. Authors Zhou F. et al. [4], Alroomi M. et al. [17], Dhont S. et al. [21], and Wu C. et al. [22] observed that the duration of illness increases which ultimately result into progression of pneumonia and having high ferritin level in these cases.

Raised ferritin after the second week of illness may indicate worsening of COVID-19 pneumonia or secondary bacterial infection which can be confirmed with procalcitonin, and it will help clinician to formulate antibiotics policy accordingly and indirectly guiding in management of these cases by assessing follow-up titers. Also, raised ferritin level documented by Abbaspour N. et al. [9] can result into hyperferritinemia causing immunosuppression and causing risk of acquiring bacterial infection as seen with COVID-19 pneumonia cases with concurrent steroid use in these cases. Authors Alroomi M. et al. [17] and Marco Meloni et al. [24] documented usefulness of procalcitonin with other inflammatory markers and help in guiding treatment approach to curtail underlying bacterial infection and prevent mortality in intensive care units.

### Correlation of BIPAP use with ferritin level in COVID-19 pneumonia cases (*n* = 1000)

In present study, BIPAP/NIV requirement during course of COVID-19 pneumonia in critical care setting has significant association with ferritin level; cases received BIPAP/NIV during hospitalization were documented normal and abnormal ferritin level in 155/44 and 165/235 cases, respectively (*p* < 0.00001).

We have observed that ferritin level has very well correlation with the requirement of BIPAP/NIV, high-flow nasal cannula oxygen supplementation, and invasive mechanical ventilation in ICU setting. Authors Allen M. et al. [15], Zhou P. et al. [16], and Alroomi M. et al. [17] observed in their study that high ferritin level is predictor of requirement of mechanical ventilation and poor predictor of outcome especially when ferritin level is > 1000 ng/ml. Authors Zhou F. et al. [4] and Rasyid H. et al. [25] observed that addition of neutrophil to lymphocyte ratio with ferritin is best predictor of ICU requirement and indirect useful marker of ventilatory support requirement in these cases. Del Valle et al. [18], Montecino-rodriguez E. et al. [20], and Wu C. et al. [22] documented correlation of ferritin level with mechanical ventilation requirement in intensive care unit. Rational for this, high ferritin and its strong association with extreme inflammatory burden and these patients have propensity to land in to hyperkinetic cytokine stimulation syndrome.

### Correlation of oxygen saturation at entry point and ferritin level in COVID-19 pneumonia cases (*n* = 1000)

In present study, ferritin level has significant association with oxygen saturation in COVID-19 pneumonia cases; cases with oxygen saturation > 90%, 75–90%, and <75% are observed as normal and abnormal ferritin level in 110/100, 150/340, and 60/240 cases, respectively (*p* < 0.00001).

We observed that as oxygen saturation drops at entry point, ferritin level increases in significant number of COVID-19 cases. Probable mechanism for correlation of hypoxia or low oxygen saturation and increase in inflammatory markers like ferritin and CRP is underlying lung parenchymal inflammation secondary to pneumonia resulting into inflammatory burden and hypoxia go hand in hand in these cases. Berna Kömürcüoğlu et al. [13] documented similar observation in their study and mentioned regarding correlation of ferritin level with SPo2 level. Alroomi M. et al. [17] observed significant hypoxia in cases with high ferritin level above 1000 ng/ml. Rasyid H. et al. [25] observed that significant hypoxia requires ICU hospitalization in cases with raised ferritin level and abnormal neutrophil-to-lymphocyte ratio in COVID-19 pneumonia cases in the study conducted in Indonesia, similarly by Sarfaraz Samreen et al. [26] in their study in Pakistan. Authors Zhou F. et al. [4], Del Valle et al. [18], Montecino-rodriguez E. et al. [20], Dhont S. et al. [21], and Wu C. et al. [22] observed raised ferritin level will help in predicting severity of illness as many of these cases are having low oxygen saturation requiring interventions in intensive care units.

### Correlation of BIPAP/NIV initiation time at entry point and ferritin level in COVID-19 pneumonia cases (*n* = 600)

In present study, timing of BIPAP/NIV requirement during course of COVID-19 pneumonia in critical care setting has significant association with ferritin level; cases received BIPAP/NIV at entry point < 1 day, 3–7 days, and after 7 days of hospitalization were documented significance in fourfold raised ferritin level in 110/70, 150/160, and 30/80 cases, respectively (*p* < 0.00001).

We observed early initiation of BIPAP/NIV in those meeting criteria of oxygenation, as oxygen saturation less than 89% at room air during hospitalization was having beneficial effect in controlling systemic immune inflammatory syndrome which can be measured by ferritin level in follow-up, may be because of improvement in oxygenation and lung compliance after use of BIPAP/NIV, as hypoxia is an important trigger for rise in inflammatory burden by means of hypoxia-inducible transcription factor. Alroomi M. et al. [17] observed significant hypoxia in cases with high ferritin level above 1000 ng/ml and documented that these patients were benefited most with early ventilatory support of BIPAP/NIV or mechanical ventilation or high-flow nasal cannula in these cases. Authors Zhou F. et al. [4], Rasyid H. et al. [25], Del Valle et al. [18], Montecino-rodriguez E. et al. [20], and Wu C. et al. [22] observed that ferritin level at entry point is very well correlated with ventilatory support requirement in intensive care unit, many cases were having cytokine storm like presentation with raised ferritin and other inflammatory markers like IL-6 and CRP.

Other important observation in this study is as follows:

#### Correlation of abnormal ferritin level at entry point (*n* = 680) and follow-up and its correlation with post-COVID lung fibrosis

In present study, follow-up of ferritin titer during hospitalization as compared to entry point abnormal ferritin has significant association in post-COVID lung fibrosis (*p* < 0.00001), i.e., ferritin at entry point to fourfold raised cases in the presence or absence of pulmonary fibrosis was 40/170 and 360/110 cases, respectively.

The elevated levels of ferritin might be linked to the overproduction of inflammatory cytokines in severe patients with COVID-19. Cytokines fight against the microbes, but when the immune system becomes hyperactive, it can damage lung tissue. Thus, ferritin production is induced by inflammatory cytokines and by tissue destruction in patients with COVID-19, and resultant outcome is lung fibrosis. Authors Alroomi M. et al. [17], Dhont S. et al. [21], and Ho et al. [27] documented that iron causes destructive effect due to inflammation and ferroptosis, which triggers ferritin production to bind with free iron as a protective mechanism to reduce tissue damage.

These results were in accordance with the findings reported by Liu et al. [28], Yasin R. et al. [29], and Yu M. et al. [30], and Wu C. et al. [22] documented that raised titers during hospitalization and follow-up were having ongoing lung inflammation and can predict future lung fibrosis; they have documented lung fibrosis in majority of cases with abnormal ferritin level and has crucial role in predicting lung fibrosis as these cases require longer hospitalization with other interventions in intensive care units, and ferritin with other inflammatory markers like CRP and LDH serve as core inflammatory marker during entire process along with HRCT thorax to predict fibrosis in these recovered cases. Authors Ruan Q. et al. [31], Lin Z. et al. [32], Fei Zhou et al. [33], and Gandini O. et al. [34] documented that raised ferritin at entry point is the indicator of poor radiological outcome, and many of these cases were having lung fibrosis and radiological sequelae in post-COVID care setting recovered in intensive care units or history of mechanical ventilation during hospitalization.

#### Correlation of normal ferritin level (*n* = 320) at entry point and follow-up and its correlation with post-COVID lung fibrosis

In present study, follow-up of ferritin titer during hospitalization as compared to entry point normal ferritin has significant association in post-COVID lung fibrosis (*p* < 0.00001), i.e., ferritin at entry point to fourfold raised cases in the presence or absence of pulmonary fibrosis was 5/35 and 115/165 cases, respectively. In this study, a small fraction of non-severe patients developed into severe cases in the first 2 weeks after symptom onset. Therefore, healthcare institutions should also pay close attention to the mild patients, identify progressors early, and provide appropriate treatment to reduce mortality

Authors Zhou F. et al. [4], Yu M. et al. [30], and Yan L. et al. [35] documented LDH, ferritin, CRP, and lymphocytes count were utilized for COVID-19 prognostic prediction; persistent abnormality in these markers indicates state of unstoppable inflammation resulting in necrosis and resultant fibrosis, which is ultimate sequelae of ARDS either due to COVID-19 or MERS. Authors Ye Z. et al. [36], Xu Z. et al. [37], Fengxiang Song et al. [38], Pan F. et al. [39], and Heshui Shi et al. [40] mentioned that increased fibromyxoid stroma and organized consolidations have propensity of residual lung fibrosis, and these findings are correlated with increased inflammatory markers in their study.

#### Correlation of other variables and ferritin level in COVID-19 pneumonia cases

1. In present study, age of patient, i.e., < 50 years and > 50 years, has significant association in COVID-19 cases with normal and abnormal ferritin level (*p* < 0.00001). We have also documented gender of included cases has significant association in COVID-19 cases with normal and abnormal ferritin level (*p* < 0.010]. We have observed high ferritin level in geriatric cases and cases above 50 years of age as compared to young patients and significant difference in gender; non-severe patients who progressed to severe cases were older and had a higher probability of having advanced underlying disease, which can be missed if cytokine level cannot be monitored in follow-up. Similarly, authors Zhou F. et al. [4], Ma A. et al. [11], Dahan et al. [14], Allen M. et al. [15], Zhou P. et al. [16], Alroomi M. et al. [17], Rasyid H. et al. [25], Del Valle et al. [18], Sarfaraz Samreen et al. [26], Montecino-rodriguez E. et al. [20], Ho et al. [27], and Wu C. et al. [22] observed role of ferritin in predicting severity in geriatric cases in their study.
2. In present study, comorbidity as diabetes mellitus, COPD, hypertension, IHD, and obesity has significant association in COVID-19 cases with normal and abnormal ferritin level (*p* < 0.00001). We observed high ferritin level in comorbid cases being its association with cases with advanced age and more inflammatory burden in these cases as compared without comorbidity. Authors Zhou F. et al. [4], Ma A. et al. [11], Dahan et al. [14], Allen M. et al. [15], Zhou P. et al. [16], Alroomi M. et al. [17], Rasyid H. et al. [25], Del Valle et al. [18], Sarfaraz Samreen et al. [26], Montecino-rodriguez E. et al. [20], and Wu C. et al. [22] observed role of ferritin in predicting severity in cases with comorbidities like DM, HTN, IHD, CKD, and COPD. Huang S. et al. [19] observed low serum ferritin in their cases with hypertension.

## Conclusions

Ferritin is easily available, sensitive and reliable, cost-effective, and universally acceptable inflammatory marker in COVID-19 pandemic. Robust data of ferritin is available in bacterial infection, and it can be utilized in this COVID-19 pneumonia pandemic for initial assessment before planning of treatment in indoor setting in comparison with other inflammatory markers and CT severity.

Ferritin has a very crucial role in COVID-19 pneumonia in predicting severity of illness; especially, follow-up titers have significant role in step-up or step-down interventions in critical care setting. Correlating ferritin with variables like duration of illness, oxygenation status, and timing of BIPAP/NIV at entry point is important to have satisfactory treatment outcome.

Ferritin titer has significant associations with predicting progression of pneumonia, as proportionate number of pneumonia cases with mild variety on CT thorax and normal initial ferritin has progressed to critical course, and we have documented follow-up titers have played crucial role with other inflammatory markers, and many times in the second week of illness, rising titers indicate nosocomial bacterial infection and target therapy accordingly.

Ferritin titer can help in predicting progression of COVID pneumonia and in assessing risk of post-COVID lung fibrosis; also, follow-up of titers in suspected lung fibrosis case can be monitored in underlying lung inflammation with this easily available marker.
